# Prostate-Specific Membrane Antigen Positron Emission Tomography/Computed Tomography-Derived Radiomic Models in Prostate Cancer Prognostication

**DOI:** 10.3390/cancers16101897

**Published:** 2024-05-16

**Authors:** Linda My Huynh, Shea Swanson, Sophia Cima, Eliana Haddadin, Michael Baine

**Affiliations:** 1 Department of Radiation Oncology, University of Nebraska Medical Center, Omaha, NE 68105, USA; lindamh@uci.edu (L.M.H.); sophia.cima@unmc.edu (S.C.); 2 Department of Urology, University of California, Irvine, CA 92868, USA; ehaddadi@hs.uci.edu

**Keywords:** prostate cancer, radiomics, personalized medicine, artificial intelligence

## Abstract

**Simple Summary:**

The contemporary development of radiomics offers an opportune methodology for the interpretation of prostate-specific membrane antigen (PSMA) positron emission tomography/computed tomography (PET/CT). While both technologies are relatively new for consideration of clinical integration, the present exploration seeks to review current literature on their intersection. Review of twenty-three peer-reviewed articles revealed promising results for the use of PSMA PET/CT-derived radiomics in the prediction of biopsy Gleason score, adverse pathology, and treatment outcomes for prostate cancer (PC). Clinical integration of these findings, however, are limited by lack of biologic validation and reproducible methodology.

**Abstract:**

The clinical integration of prostate membrane specific antigen (PSMA) positron emission tomography and computed tomography (PET/CT) scans represents potential for advanced data analysis techniques in prostate cancer (PC) prognostication. Among these tools is the use of radiomics, a computer-based method of extracting and quantitatively analyzing subvisual features in medical imaging. Within this context, the present review seeks to summarize the current literature on the use of PSMA PET/CT-derived radiomics in PC risk stratification. A stepwise literature search of publications from 2017 to 2023 was performed. Of 23 articles on PSMA PET/CT-derived prostate radiomics, PC diagnosis, prediction of biopsy Gleason score (GS), prediction of adverse pathology, and treatment outcomes were the primary endpoints of 4 (17.4%), 5 (21.7%), 7 (30.4%), and 7 (30.4%) studies, respectively. In predicting PC diagnosis, PSMA PET/CT-derived models performed well, with receiver operator characteristic curve area under the curve (ROC-AUC) values of 0.85–0.925. Similarly, in the prediction of biopsy and surgical pathology results, ROC-AUC values had ranges of 0.719–0.84 and 0.84–0.95, respectively. Finally, prediction of recurrence, progression, or survival following treatment was explored in nine studies, with ROC-AUC ranging 0.698–0.90. Of the 23 studies included in this review, 2 (8.7%) included external validation. While explorations of PSMA PET/CT-derived radiomic models are immature in follow-up and experience, these results represent great potential for future investigation and exploration. Prior to consideration for clinical use, however, rigorous validation in feature reproducibility and biologic validation of radiomic signatures must be prioritized.

## 1. Introduction

Prostate cancer (PC) continues to be the most common non-cutaneous malignancy among men [1,2,3], demanding continuous advancements in diagnostic and prognostic methodologies. Over the past few decades, significant strides have been made in the realm of medical imaging techniques, aiding early detection, precise localization, and improvement in risk stratification for PC patients. Among these is the prostate-specific membrane antigen (PSMA) positron emission imaging and computerized tomography (PET/CT), which was approved by the Food and Drug Administration (FDA) in December 2020 and May 2021 for use with 68-Gallium (68-Ga) and piflufolastat (F18) in PC patients, respectively [4]. Since then, several systematic reviews have confirmed the PSMA PET/CT to more accurately detect the extent of disease [5], lymph node involvement [6,7], and distant metastases in patients newly diagnosed with PC [5,7,8].

Beyond its diagnostic impact, however, the integration of PSMA PET/CT into the PC clinical care pathway also represents potential for leveraging advanced data analysis techniques for prognostication. Among these is the use of radiomics, a computer-based method of extracting and quantitatively analyzing subvisual imaging characteristics [9]. Not only can these radiomic features (i.e., textural, morphological, functional, or statistical) be used to characterize subvisual patterns in tumor morphology and the microenvironment [10], but they can also be aggregated into models to predict long-term treatment outcomes [11,12]. Within the context of PC, similar models utilizing multiparametric magnetic resonance imaging (mpMRI) [1,2] and ultrasound (U/S) [13] have also demonstrated potential to facilitate disease-tailored treatment planning. In this regard, the present review seeks to summarize the current literature on the use of PSMA PET/CT-derived radiomics in the diagnosis, staging, and treatment of localized PC.

## 2. Methods

A stepwise literature search of publications from 2017 to 2023 was performed. A search of Medical Literature Analysis and Retrieval System Online (MEDLINE) databases was completed utilizing the following keywords and combination(s) thereof: [radiomics] with/without [prostate cancer] or [prostate], interchanged with [PSMA] and/or [PSMA PET] and/or [PSMA PET/CT]. This yielded 40 articles. Non-English publications, review articles, editorials, and commentaries were excluded, but the reference list of each was searched to ensure the inclusion of all relevant studies.

Utilizing the following stepwise methodology, studies were reviewed by the study team for inclusion and exclusion criteria defined a priori. First, the titles and abstracts were screened such that non-relevant studies pertaining to other imaging techniques and/or other diagnoses were excluded. Second, full manuscripts were reviewed for their study populations and/or outcome measures. All authors independently agreed on the selection of eligible studies and achieved consensus of included studies. Data on the number of subjects, outcome measures, image series used, radiotracers, feature selection, region of interest, and model validation were systematically extracted from each article and summarized in Table 1, Table 2, Table 3 and Table 4. Studies were not excluded based on type of radiotracer used but, to ensure standardization, the International Society of Urological Pathology (ISUP) guidelines on Gleason score (GS) [14], National Comprehensive Center Network (NCCN) and American Urological Association (AUA) guidelines for risk group stratification [15,16], and Prostate Imaging Reporting and Data System version 2 (PI-RADS v2) were utilized [17].

## 3. Results

### 3.1. Study Selection

**Figure 1:**
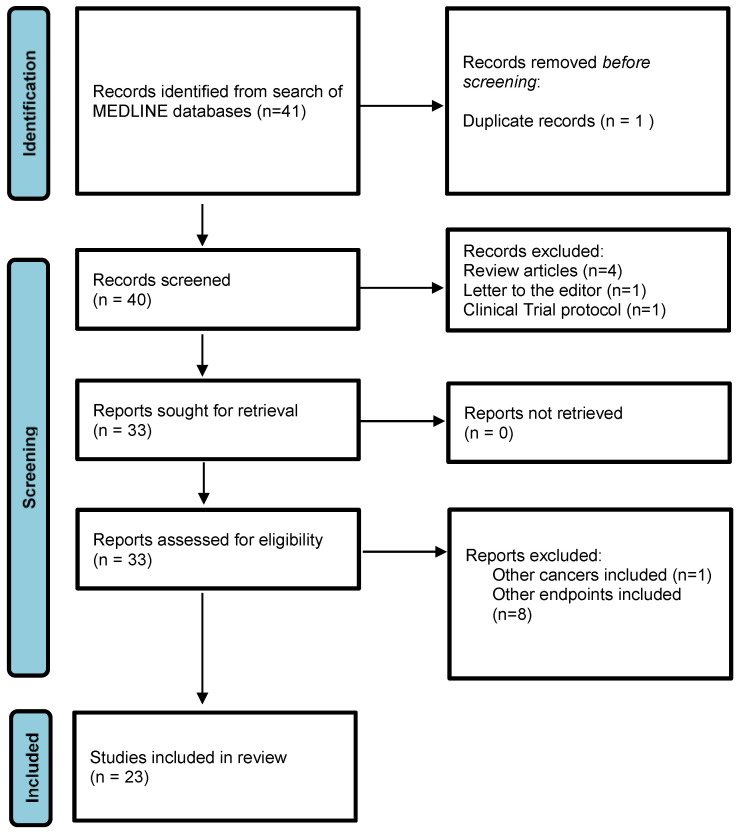
Study selection flow diagram.

Forty publications were first identified and screened through a literature search of the MEDLINE journals via a PubMed interface. Of these, 4 review articles, 1 letter to the editor, and 1 clinical trial protocol were excluded, leaving 33 records for title and abstract review. In this process, two additional records were excluded for the inclusion of diseases outside of prostate cancer and eight were excluded for endpoints related to imaging quality (*n* = 1), scan reliability across series (*n* = 3), molecular imaging (*n* = 3), and feature stability (*n* = 1). After all inclusion and exclusion criteria were satisfied, 23 articles remained and were reviewed herein. Figure 1 depicts a schematic of study selection and inclusion/exclusion criteria. 

### 3.2. Description of Studies

Of the 23 included studies, all were published between 2017 and 2022, with the majority (*n* = 20, 87.0%) published in 2020 or later. There were no results of PSMA PET/CT-derived radiomics work prior to 2017. Additionally, most explorations utilized imaging achieved exclusively with 68-Ga-PSMA radiotracers (*n* = 18, 78.3%), with the second majority exclusively utilizing Fluoride-18-PSMA-1007 (*n* = 4, 17.4%). One study utilized a combination of both 68-Ga-PSMA and Fluoride-18-PSMA.

PC diagnosis, prediction of biopsy GS, prediction of adverse pathology, and treatment outcomes were the primary endpoints of four (17.4%), five (21.7%), seven (30.4%), and seven (30.4%) studies, respectively. Of those predicting treatment outcomes, treatment response following radiation therapy (*n* = 1), biochemical recurrence following surgery (*n* = 2), response following 177Lu-PSMA therapy (*n* = 1), post-ADT PSA levels (*n* = 1), and overall survival (*n* = 2) were indicated to be the primary endpoints. Finally, of all studies included, only two (8.3%) included external validation and two others (8.3%) included a multi-institutional cohort of patients.

#### 3.2.1. Prediction of Prostate Cancer Diagnosis

Table 1 summarizes the four studies utilizing PSMA PET/CT-derived radiomics models in initial PC diagnosis [18,19,20,21]. Of these, three (75.0%) and one (25.0%) utilized the 68-Gallium PSMA-11 PET/CT [19,20,21] and PSMA-1007 PET/CT [18], respectively. Two (50%) studies utilized intraprostatic lesions as the region of interest, with the outcome measure predicting lesions positive for PC [19,20]; the remaining two studies (50%) utilized the full prostate as the region of interest, predicting PC risk group classification [18] and bone metastases [21] as their primary outcome measures. Receiver operator characteristic curve area under the curve (ROC-AUC) ranged from 0.85 to 0.925. Furthermore, Zang and colleagues found their radiomic model to perform significantly better in predicting positive PC lesions when compared to the radiologist’s assessment (radiomic model’s ROC-AUC = 0.85 vs. radiologic assessment ROC-AUC = 0.63, *p* = 0.036) [19]. While all radiomic-based models performed well, none of these studies included validation with an external cohort of patients.

#### 3.2.2. Prediction of Biopsy Results

Table 2 illustrates five studies utilizing PSMA PET/CT-derived radiomic models in PC staging [8,22,23,24,25]. Three studies [22,24,25] (66.7%) utilized 68-Ga-PSMA-11 imaging and two [8,23] (33.3%) utilized Fluoride-18-PSMA-11 imaging alterations and biopsy Gleason score [25]. Sample sizes in these studies ranged from 10 patients [25] to 173 patients [23] and final models included the highest number of features selected when compared to other published PSMA-derived radiomic models (range: 70–336 features selected). Most studies (*n* = 4, 80.0%) delineated the prostate as the region of interest during image processing, with only one [22] utilizing intra-prostatic lesions. All studies were internal validations between training and testing cohorts, or cross-validations, and external validation was not included.

ROC-AUC of the final radiomic models ranged from 0.719 to 0.84. Kesch et al. reported that lower ADC values correlated with increasing tumor aggressiveness but did not include a predictive model in their analysis [25].

#### 3.2.3. Prediction of Adverse Pathology following Radical Prostatectomy

Table 3 summarizes the seven studies utilizing PSMA PET/CT-derived radiomics in the identification and prediction of adverse pathology. Of the studies included, five (71.4%) utilized PSMA PET/CT-derived radiomics to predict adverse pathology (i.e., GS, LNI, and ECE) following RP (Table 3) [6,28,30,31]. Three (42.9%) studies reported on LNI [6,28,29], five (71.4%) reported on GS characterization [27,28,29,30], and one (14.3%) reported ECE [28]. Zamboglou et al. used both GS and LNI as their primary endpoint [29], while Cysouw et al. also included any metastasis and ECE [28]. Furthermore, Zamboglou and colleagues focused on detecting “visually undetectable” lesions that were initially missed upon scanning. Solari, Tu, and Papp et al. utilized PSMA PET/MRI-derived radiomics to predict adverse pathology (specifically, through GS characterization) following RP. Of the seven studies, Papp et al. was the only one to use a dual tracer with 68-Ga-PSMA-11 and 18-F-DCFPyL [28,30].

Within these studies, four also included secondary classification of GS risk groups [26,27,28,29,30]. Solari and colleagues compared radiomic features extracted from different imaging sequences (i.e., T1w, T2w, and ADC) and found that the PET + ADC radiomics model outperformed other double and single modalities [27]. In determining regions of interest, two studies utilized the whole prostate [27,28] and two utilized intraprostatic lesions [29,30].

Overall, the ROC-AUC of final radiomic models ranged from 0.81 to 0.86, with only Zamboglou et al. performing external validation. For the prediction of GS 7 versus ≥8, their model yielded an ROC-AUC on a training set of 0.91 and an ROC-AUC on a testing set of 0.84 [29]. The ROC-AUC on training sets ranged from 0.87 to 0.89, while the ROC-AUC on testing sets ranged from 0.85 to 0.95. Cysouw and colleagues used Random Forest for the prediction of LNI, which yielded an AUC of 0.86; additionally, they reported ECE, which yielded an AUC of 0.76 [28].

#### 3.2.4. Prediction of Prostate Cancer Recurrence

Table 4 provides a summary of the seven studies predicting PC recurrence following RP (*n* = 2, 28.6%) [2,30] and following radiation (*n* = 5, 71.4) [3,32,33,34,35]. Of these seven studies, six (86.0%) used PSMA PET/CT-derived radiomics to primarily predict biochemical recurrence (BCR), treatment response, and overall survival. Tran et al. was the only study that focused on predicting treatment response solely to ADT: patients received 3 months of ADT with 6 months remaining and had no prior RP or radiation [12].

Three studies (42.9%) reported on BCR [2,30,32], three (42.9%) reported on treatment response [3,12,33], and three (42.9%) reported on overall survival (OS) [32,34,35]. Assadi et al. used both BCR and OS as primary endpoints [32]. Within the eight studies, four (57.1%) delineated the prostate [2,12,32,34] as the region of interest, while three (42.9%) delineated intraprostatic lesions [3,27,33,35].

Overall, feature extraction and selection were most commonly performed via Random Forest [32,35] and the ROC-AUC of final radiomic models ranged from 0.698 to 0.90. Tran et al. identified specific radiomic features that helped distinguish responders to ADT in all three zones of the prostate (*p* = 0.012–0.038), but no aggregated model was supplied [12]. Similarly, Moazemi and colleagues found that features representing SUVmin, kurtosis, calculated RS, and SUVmean were statistically significant (*p*-value < 0.05) in predicting OS [35].

## 4. Discussion

Given the recent approval and addition of the PSMA PET/CT scan to the PC clinical care pathway, the progression to PSMA PET/CT-derived radiomics in risk stratification and personalized management of PC is a logical and potentially transformative advancement. Much of the groundwork for radiomic-related machine learning models has been established with years of investigation via multiparametric MRI imaging [1,2,24,36] and, given this, it is unsurprising that all the articles reviewed herein were accomplished within the last three years following FDA approval of the 68-Ga-PSMA PET/CT in PC patients [4]. Keeping in mind that image interpretation and segmentation is limited by interobserver variability, the use of radiomic models has the potential to enhance diagnostic performance. Even further, recent advances in automated segmentation [23] and image preprocessing [27] may further facilitate efficient clinical integration should an adequately validated model be achieved.

A clear focus of PSMA PET/CT-derived radiomics has been in prediction of surgical pathology [6,26,27,28,29,30,31], treatment outcomes [2,3,7,12,30,32,33,34,35], progression, or survival [32,34,35]. This is in stark contrast to several recent reviews of mpMRI-derived radiomic models [1,2,36], which have concentrated on the initial diagnosis and staging of PC. While this is perhaps partially due to differences in indication between mpMRI imaging versus PSMA PET/CT scans, it may also be indicative of anticipated clinical utility and potential integration into patient management. The prediction of long-term outcomes such as recurrence, progression, and survival offer the potential to alter patient management and encourage multimodal treatment. Of the 23 investigations included in the present review, 18 (78.3%) utilized PSMA PET/CT-derived radiomics for such predictions; even further, a higher proportion of recent investigations (2022 and after) concentrated on these outcomes, as compared to earlier investigations aiming to supplement Gleason score risk stratifications and the identification of positive intraprostatic lesions on prostate biopsy [25,29].

Overall, most of the included studies illustrated good-to-excellent ROC-AUC values, highlighting the potential for PSMA PET/CT-derived radiomics. Compared to previous review articles reporting on mpMRI-derived radiomic models, the studies yielded higher ROC-AUC values with sensitivity ranging from 64 to 82% and specificity ranging from 73 to 82%. Even further, a few studies compared PSMA PET/CT-derived radiomic models to standard clinical risk stratification tools and found significant improvement with the inclusion of radiomic features [19,32]. Lastly, one exploration by Assadi in 2022 constructed a clinicopathologic-radiomic nomogram to predict biochemical recurrence following 177Lu-PSMA treatment, yielding an impressive ROC-AUC of 0.827 [32].

While these studies represent promising avenues for the use of PSMA PET-CT-derived radiomics in predicting high-risk features, it is unclear whether radiomics can outperform the visual assessment of PSMA PET/CT by experienced radiologists. Of the four studies on the prediction of PC diagnosis, for example, only two (50%) performed a direct comparison between the performance of the radiomic model with that of the radiologists’ reading [19,21]. The radiomic model by Hinzpeter et al., for example, detected only 90% of the PSMA-avid metastases identified by radiologists [21]. While this leaves the percentage of newly detected metastases to be desired, direct comparison between radiomics and gold standard procedures ought to be considered in future study designs.

As the field of radiomics continues to mature, it is clear that the direct prediction of treatment outcomes is a topic of close exploration. However, given the long natural life history of PC and long follow-up needed to provide reliable clinical information, many of the papers included herein elected to utilize short-term metrics closely correlated with recurrence, progression, and survival outcomes. While many of these metrics are established strong predictors, it is important to note that they are not perfect 1:1 predictors. As such, we caution that the interpretation of models reporting excellent correlation with these interim predictors should be considered within this context. Furthermore, as the methodology of radiomics is a pipeline of operations and each operation can be modified, subsequent models are sensitive to these modifications and investigations on radiomic variability, robustness, and reproducibility are required during the interpretation of results [37,38,39]. The literature included in this review is of no exception and, as such, conclusions are limited by small sample sizes [21,22,24,25], the inclusion of single institutions without external validation, and high variability in image preprocessing and during the identification of regions of interest. Cross-validation, resampling, and multiple segmentations were employed by many of these studies [2,3,6,7,18,19,20,22,26,27,28,30,33], but the fragility of radiomic features and the variability between models ought best to be addressed via external validation and reproducibility. Despite these concerns, however, of the 23 studies included in the present review, only 2 (8.7%) performed external validation of the radiomic model in an external cohort of patients. As we consider future efforts for study design and validation, we also caution against the inclusion of different PSMA tracers in these studies, as the added layer of variability in tracer sensitivity and specificity may influence results. Given the relative infancy of PSMA PET/CT-derived radiomics in PC, stepwise improvements in study design will facilitate increased generalizability, validation, and clinical integration.

Finally, as exemplified by the present review, the traditional radiomics pipeline proposes an imaging-derived signature of disease outcome and seeks to subsequently develop and validate a predictive model in an independent training set. Although these models may prove to be robust predictors of a given outcome, clinical integration demands further evidence of a biologic relationship and/or molecular mechanism. As the field of radiomics continues to grow and develop, intersectionality with histology [39,40], pathology [41,42], and genomics [43,44] offers high potential for biologic validation and improved clinical interpretability of radiomic models. Correlation with local pathologic analysis, for example, can provide a direct comparison of quantitative, pathologic features to explain structural characteristics underlying radiologic textures. Even further, correlations with genomic data can provide a link to the molecular pathways underlying tumor biologic characteristics. These explorations would not only facilitate a comprehensive understanding of the interplay between macroscopic imaging features and microscopic tissue properties, but they can also facilitate the translation of radiomic findings into clinically actionable insights.

## 5. Conclusions

Given the recent approval and integration of PSMA PET/CT into the PC clinical care pathway, PSMA PET/CT-derived radiomics offers high potential for improved PC risk stratification and prediction of treatment response. However, while current studies show promise in predicting PC diagnosis, biopsy features, pathology, and treatment outcomes, these explorations are limited by small sample sizes and a lack of external validation. As the field of radiomics continues to mature, concerted efforts to enhance the reproducibility of radiomics and biologically validate these radiomic models must be pursued prior to clinical integration.

## Figures and Tables

**Table 1:** Summary of studies utilizing PSMA PET/CT-derived radiomic models in PC diagnosis.

Author	Year	*n*=	Radiotracer	Outcome Measure	Feature Selection	Region of Interest	Model Validation	Results
Leung [18]	2022	*n* = 267	Fluoride-18-PSMA-1007	PSMA-RADS and PC classification	6 features selected	Prostate	Cross-validation between training, testing, and validation data sets	AUC = 0.87 and 0.90 for lesion-level and patient-level PSMA-RADS classification. AUC = 0.92 and 0.85 for lesion-level and patient-level PC classification
Zang [19]	2022	*n* = 125	68-Ga-PSMA-11	Prediction of intraprostatic lesions	944 features extracted/ 9 features selected	Intraprostatic lesions	Cross-validation *n* = 87 in training group, *n* = 38 in testing group	Radiomics model AUC = 0.85 vs. AUC = 0.63 for radiologists’ assessment (*p* = 0.036); Radiomics model vs. radiologist sensitivity AUC = 0.84 vs. AUC = 0.74 (*p* = 0.002)
Yi [20]	2022	*n* = 100	68-Ga-PSMA-11	Diagnosis of intraprostatic lesions invisible on PET	1781 features extracted/ 10 features selected	Intraprostatic lesions	Cross-validation *n* = 64 in training set, *n* = 36 in testing set	3 radiomic models with AUC = 0.903, 0.856, and 0.925 (*p* = 0.007, 0.045, and 0.005, respectively)
Hinzpeter [21]	2021	*n* = 67	68-Ga-PSMA-11	Diagnosis of metastatic bone cancer from PC	1218 features extracted/ 11 features selected	Prostate	Internal validation with the original non-augmented data set	90% diagnostic accuracy, 91% sensitivity, and 88% specificity

**Table 2:** Summary of studies utilizing PSMA PET/CT-derived radiomic models in PC staging via biopsy.

Author	Year	*n*=	Radiotracer	Outcome Measure	Feature Selection	Region of Interest	Model Validation	Results
Chan [22]	2023	*n* = 19 patients	68-Ga-PSMA-11	Tumor location and grading (Grade Group scores of ≥3 for high grade and ≤2 for low grade)	75 features selected/ 10 features analyzed	Intra-prostatic lesions (IPLs)	Cross-validation with Random Forest Classifier and Support Vector Classifier	Overall model, AUC = 0.890
Wang [8]	2022	*n* = 161 patients	Fluoride-18-PSMA-1007	PSA level, Gleason score, metastasis status	944 features selected/ 30 features analyzed	Prostate	Internal validation with training and test cohorts	Gleason score model ROC-AUC = 0.719, *p* < 0.01
Yao [23]	2022	*n* = 173 patients	Fluoride-18-PSMA-1007	Gleason score, extracapsular extension, vascular invasion	70 features selected/ 10 features analyzed	Prostate	Internal validation with training and test cohorts	Best model: 40–50% SUVmax AUC 0.81, *p* < 0.001
Feliciani [24]	2022	*n* = 56 scans	68-Ga-PSMA-11	ISUP grade	218 features selected/29 features analyzed (for PET/CT model) 218 features selected/87 features analyzed (for MRI model)	Prostate	Internal validation with training and test cohorts	MRI AUC = 1.00 in testing and training groups MRI + PET/CT AUC = 1.00 in training group
Kesch [25]	2018	*n* = 10	68-Ga-PSMA-11	Chromosomal copy number alterations (CNAs), Gleason score	336 features extracted	Prostate (genomic index lesions)	N/A	Lower ADC values correlate with increasing tumor aggressiveness

**Table 3:** Summary of studies utilizing PSMA PET/CT-derived radiomic models in identification of adverse pathology.

Author	Year	*n*=	Radiotracer	Outcome Measure	Feature Selection	Region of Interest	Model Validation	Results
Ghezzo [26]	2023	*n* = 47 patients (PET/CT or PET/MRI)	68-Ga-PSMA-11	Postsurgical GS	154 features selected/ 2 features analyzed	Prostate	Cross-validation	ECE AUC = 0.76 ± 0.12, *p* < 0.01”
Solari [27]	2022	101 patients	68-Ga-PSMA-11	Postsurgical GS (ISUP grades 1–3, grade 4, and grade 5)	480 features selected/ 48 features analyzed	Prostate	External validation cohort (52 patients)	Radiomics-based machine learning model: LNI AUC = 0.86 ± 0.15, *p* < 0.01
Cysouw [28]	2020	76 patients	18-F-DCFPyL	LNM, presence of metastasis, GS, ECE	133 features extracted/ 86 features analyzed (analysis 2), 56 features analyzed (3a), 1 feature analyzed (3b)	Prostate	Internal validation by retrospective cohort (40 patients)	QSZHGE feature GS: training-AUC = 0.91 and testing-AUC = 0.84; *p* < 0.01
Zamboglou [29]	2020	72 patients	68-Ga-PSMA-11	ISUP grade, undetected lesions	Spearman’s correlation coefficients, Wilcoxon (1), Mann-Whitney U test (2 and 3)	Intraprostatic tumor lesions	5-fold cross-validation	QSZHGE feature LN status: training-AUC = 0.87 and testing-AUC = 0.85; *p* < 0.01
Papp [30]	2020	52 patients	68-Ga-PSMA-11 and 18-F-FMC	low vs. high lesion risk, BCR, OPR	RaCaT software	Intraprostatic tumor lesions	6-fold cross-validation with training (67 patients) and testing (34 patients) cohorts	Distal metastasis AUC = 0.86 ± 0.14, *p* < 0.01
Peeken [6]	2020	80 patients	68-Ga-PSMA-11	LNM	156 features extracted	Intraprostatic tumor lesions	10-fold cross-validation with training cohort (47 patients)	Best model (radiomics-combined): testing-AUC = 0.95 and training-AUC = 0.89, *p* = 0.0035
Zamboglou [31]	2019	20 patients	68-Ga-PSMA-11	GS 7, ≥8 and pelvic LNM	ComBatHarmonization and LASSO	Intraprostatic tumor lesions	External testing cohort (33 patients)	LBP features showed highest contribution to model performance

**Table 4:** Summary of studies utilizing PSMA PET/CT-derived radiomic models in the identification of treatment response.

Author	Year	*n*=	Radiotracer	Outcome Measure	Feature Selection	Region of Interest	Model Validation	Results
Spohn [2]	2023	99 patients	68-Ga-PSMA-11	BCR after salvage radiation therapy	104 features extracted	Prostate	Nested cross-validation multi-center study	Radiomic signature AUC 0.73, *p* < 0.001
Assadi [32]	2022	33 patients (2517 pathological hotspots)	68-Ga-PSMA-11	BCR after 177Lu-PSMA and overall survival	Mutual information feature selection	Prostate	Multi-center study	Combined clinical and radiomic signature AUC 0.63; improved sensitivity (0.26 to 0.78)
Tran [12]	2022	35 patients (70 scans)	68-Ga-PSMA-11	Treatment response to ADT	119 features extracted	Prostate (3 zones)	N/A	7 features in zone 1 distinguished responders to ADT 2 features classifying nodal disease: AUC 0.698, *p* < 0.001
Moazemi [33]	2021	83 patients (2070 pathological hotspots)	68-Ga-PSMA-11	Overall survival after 177Lu-PSMA	SUVmax: 80 features analyzed (zone 1), 21 (zone 2), 3 (zone 3)	Intraprostatic lesions	5-fold cross-validation with training and testing cohort	Higher T2 interquartile range showed longer OS, *p* = 0.038 2 features in zone 2; *p*-value 0.018–0.34
Roll [34]	2021	21 patients	68-Ga-PSMA-11	PSA response and overall survival	PyRadiomics	Prostate	Unbalanced cohort: training *n* = 56 patients in validation; *n* = 27 in testing cohorts	2 features in zone 3; *p*-value 0.012–0.19
Papp [30]	2020	52 patients	68-Ga-PSMA-11 and 18-F-FMC	low vs. high lesion risk, BCR, OPR	ExtraTrees	Intraprostatic tumor lesions	10-fold cross-validation with 9:1 training–testing cohort	3 features classifying tumor relapse: AUC 0.726, *p* < 0.002
Acar [3]	2019	75 patients (126 scans)	68-Ga-PSMA-11	Metastasis status	SVM	Intraprostatic lesions	N/A	Highest accuracy prediction biochemical response: T2w AUC 0.83
